# Association of blood cadmium levels with all-cause and cause-specific mortality among adults with non-alcoholic fatty liver disease: a prospective cohort study

**DOI:** 10.3389/fpubh.2025.1573760

**Published:** 2025-04-04

**Authors:** Congxi Xu, Zhi Li, Shirui Hao, Jian Zhang, Jinlong Li, Kuopeng Liang, Xiaojuan Wang, Yi Zhang, Guangyuan Zhao, Mengyun Bai, Dengxiang Liu, Jitao Wang

**Affiliations:** ^1^Hebei Provincial Key Laboratory of Cirrhosis and Portal Hypertension, Xingtai People’s Hospital of Hebei Medical University, Xingtai, Hebei, China; ^2^Graduate School of Hebei Medical University, Shijiazhuang, Hebei, China; ^3^Department of Infection Management, North China Healthcare Group Xingtai General Hospital, Xingtai, Hebei, China; ^4^Hepatopancreatobiliary Center, Beijing Tsinghua Changgung Hospital, School of Clinical Medicine, Tsinghua University, Beijing, China

**Keywords:** cadmium exposure, non-alcoholic fatty liver disease, NHANES, mortality, prognosis, United States

## Abstract

**Background:**

Cadmium (Cd) accumulates in the body over time, damaging organs such as the liver, kidneys, and brain. Some researchers have suggested that elevated blood Cd levels may contribute to the onset and progression of nonalcoholic fatty liver disease (NAFLD). However, only a few studies have explored the relationship between Cd exposure and long-term health outcomes in patients with NAFLD. This study aimed to evaluate the predictive value of blood cadmium levels for mortality risk in patients with NAFLD.

**Methods:**

This study analyzed data from 13,450 patients with NAFLD in the National Health and Nutrition Examination Survey (NHANES) database, covering the years 1999 to 2018. Patients were categorized into three groups based on their blood Cd levels. The relationship between blood cadmium concentrations and all-cause, cardiovascular, and cancer mortality in NAFLD patients was assessed using Cox proportional hazards regression while accounting for potential confounders. Results were visualized using Kaplan–Meier and restricted cubic spline (RCS) curves. Stratified analyses were performed for validation of the robustness of the results.

**Results:**

After adjusting for all covariates, blood Cd levels were positively associated with all-cause, cardiovascular, and cancer mortality in patients with NAFLD, showing a significant linear dose–response relationship. Specifically, for each unit increase in Log-transformed blood cadmium concentration, the risk of all-cause mortality increased by 191% (HR = 2.91, 95% CI: 2.39–3.53); cardiovascular mortality risk increased by 160% (HR = 2.6, 95% CI: 1.80–3.76); and cancer mortality risk increased by 279% (HR = 3.79, 95% CI: 2.54–5.65). Stratified analysis confirmed the robustness of these findings.

**Conclusion:**

Our study suggests that high Blood Cd levels adversely affect the prognosis of patients with NAFLD. Individuals with NAFLD should be aware of Cd exposure and take preventive measures. Moreover, stricter environmental protection policies may be necessary to reduce Cd exposure.

## 1 Background

Nonalcoholic fatty liver disease (NAFLD) represents a significant contributor to the prevalence of chronic liver disorders (1, 2). It is a metabolic disorder characterized by the presence of fatty degeneration in ≥5% of liver cells without other obvious causes, such as excessive alcohol consumption or viral hepatitis (3). The prevalence of NAFLD worldwide is approximately 25%–30% and is steadily increasing (4, 5). NAFLD is associated with multiple systemic metabolic disturbances, putting patients at increased risk for cancer, cardiovascular diseases, and cirrhosis (6). Currently, there is no specific medication for NAFLD; treatment primarily focuses on modifying metabolic risk factors to improve long-term outcomes (7). NAFLD imposes a significant economic burden and has emerged as a major global public health issue (8, 9). Therefore, identifying biomarkers that may be predictive of the prognosis of patients with NAFLD is crucial.

Cadmium (Cd) is a common environmental pollutant found in industrial production processes, contaminated rice and shellfish, batteries, pigments, cosmetics, and hair dyes. It enters the human body through food, air, soil, drinking water, and other pathways (10, 11). Cd accumulates in the liver, inducing extensive liver damage and ultimately leading to NAFLD (12, 13). It may promote the progression of NAFLD by inhibiting mitochondrial transfer and increasing intracellular lipid accumulation (14). Previous studies have reported the adverse effects of Cd exposure on the prognosis of the general U.S. population, postmenopausal women, and patients with hypertension (15–17). Some researchers have examined the relationship between blood Cd levels and NAFLD (18, 19). A recent review summarized existing epidemiological and laboratory research findings, suggesting an association between cadmium exposure and an increased risk of NAFLD as well as changes in liver damage markers (20). Furthermore, studies have indicated that early-life exposure to cadmium increases the risk of cognitive impairment in adulthood, potentially related to corticosterone responses and immune dysregulation (21, 22). Maternal exposure to cadmium may be associated with the development of NAFLD in offspring. Additionally, experiments in mice have confirmed that early-life exposure to cadmium induces the occurrence of liver tumors (23). However, there is a lack of conclusive evidence regarding the effect of blood cadmium levels on the prognosis of patients with NAFLD.

Therefore, this study aimed to provide evidence of the association between blood cadmium levels and the risk of mortality in patients with NAFLD using a large, nationally representative dataset from the National Health and Nutrition Examination Survey (NHANES) and the National Death Index (NDI). This research may facilitate timely and appropriate preventive and medical interventions to improve the prognosis of patients with NAFLD.

## 2 Methods

### 2.1 Study population

The NHANES, overseen by the Centers for Disease Control and Prevention (CDC), is a nationally representative survey aimed at evaluating the health and nutritional status of both adults and children in the United States. Informed consent from the National Centre for Health Statistics Institutional Review Board was obtained from all participants. Data from NHANES have been made publicly available online biennially since 1999 (24). Given the use of pre-existing de-identified data from NHANES and the waiver of the need for informed consent for this study, the National Center for Health Statistics Ethics Review Board granted an exemption for this research.

**Figure 1:**
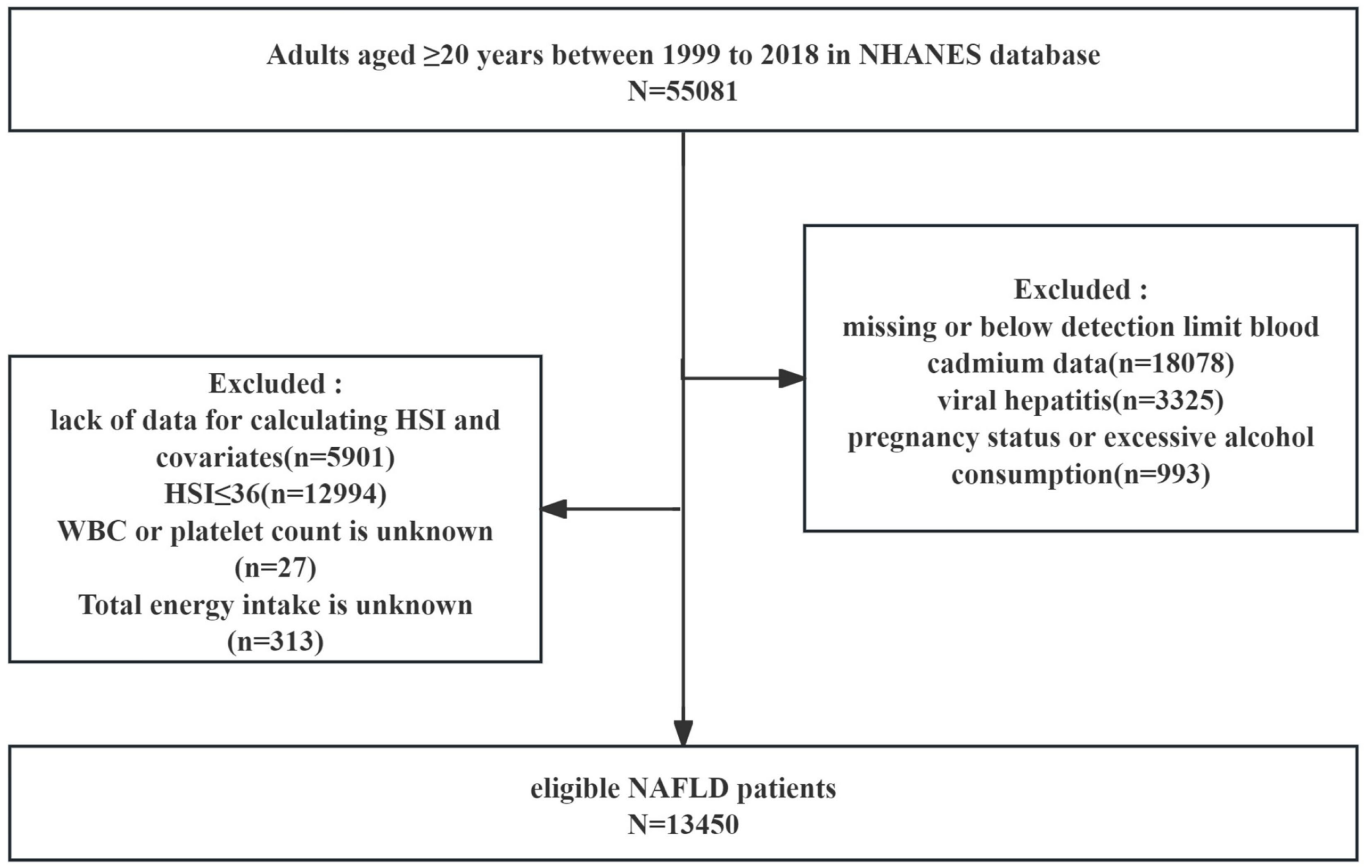
Flow diagram of patient inclusion from the NHANES database.

We included 55,081 adults aged ≥ 20 years from NHANES 1999–2018 in the analysis. Exclusion criteria were as follows: (a) missing or below-detection-limit blood cadmium data (*n* = 18,078); (b) viral hepatitis (*n* = 3,325); (c) pregnancy or excessive alcohol consumption (*n* = 993); (d) lack of data for calculating hepatic steatosis index (HSI) and covariates (*n* = 5,901); (e) HSI ≤ 36 (*n* = 12,994); (f) White blood cell (WBC) count or platelet count is unknown (*n* = 27); and (g) Total energy intake is unknown (*n* = 313). Ultimately, 13,450 eligible participants were included in the analysis (Figure 1).

### 2.2 Cadmium exposure

Whole blood specimens were transported to the Laboratory Science Department, the National Center for Environmental Health, and the CDC for analysis. Blood Cd levels were assessed utilizing atomic absorption spectrometry from 1999 to 2002, and subsequently measured using inductively coupled plasma mass spectrometry from 2003 to 2018. Detailed information on the experimental methods and quality assurance measures can be found online (24). A natural logarithmic (log) transformation was applied to the blood Cd concentrations to reduce data skewness, as the distribution of blood Cd levels was highly skewed. This transformation helped normalize the data, allowing for more accurate statistical modeling and interpretation.

### 2.3 Nonalcoholic fatty liver disease

NAFLD was defined as HSI > 36 (25). The calculation formula for HSI was as follows: $\begin{array}{l} HSI=8\times \left(\begin{array}{l} \mathrm{alanine aminotransferase}\;\left(\mathrm{ALT}\right)/\\ \mathrm{aspartate aminotransferase}\;\left(AST\right)\;\mathrm{ratio} \end{array}\right)+\\ \mathrm{body mass index}\;\left(BMI\right)\;\\ \left(+2\;\mathrm{for females;}+2\;\mathrm{for diabetes}\right)\; \end{array}$ (26).

### 2.4 Mortality ascertainment

NHANES data were linked to mortality data from the NDI. This study followed participants from participating in the survey until 31 December 2019. The determination of causes of death was conducted based on the International Classification of Diseases, Tenth Revision (ICD-10). Outcomes included all-cause, cardiovascular, and cancer-related mortalities.

### 2.5 Covariate definitions

The following covariate information was collected from NHANES for analysis: age, sex, race, education, marital status, poverty income ratio (PIR), BMI, diabetes, moderate physical activity, smoking status, blood cotinine concentration, drinking status, and presence of hypertension. PIR was categorized as PIR < 1 (low income), 1 ≤ PIR ≤ 3 (medium income), and PIR > 3 (high income). Smoking status was classified as yes (lifetime smoking ≥ 100 cigarettes) or no (lifetime smoking < 100 cigarettes). Drinking status was categorized as yes (≥12 drinks per year) or no (<12 drinks per year). Diabetes was diagnosed based on the patient’s questionnaire responses, fasting blood glucose levels, and glycated hemoglobin levels. Hypertension was diagnosed on the patient’s medical history. Energy intake is defined as the total caloric intake per day. The FIB-4 index $\left(\mathrm{FIB}-4=\frac{age*AST}{\mathrm{ALT}*\;\sqrt{\mathrm{platelet}}}\right)$ is used to represent the severity of liver fibrosis in patients. A FIB-4 index of ≥2.67 indicates advanced fibrosis (27).

### 2.6 Statistical analyses

We used Cox proportional hazards regression analysis to assess the relationship between blood Cd levels in patients with NAFLD and all-cause, cardiovascular, and cancer mortality while considering possible confounders. Crude model unadjusted for covariates. Model 1 was adjusted for age, sex, race, and educational level. Model 2 included adjustments from model 1 and also considered marital status, PIR, BMI, and moderate physical activity. Model 3 was adjusted for the covariates in Model 2, plus smoking status, cotinine levels, drinking status, hypertension, diabetes, WBC, energy intake and FIB-4 index. Participants were grouped into tertiles based on blood Cd levels. Kaplan–Meier curves were drawn, and log-rank tests were performed between groups. RCS regression models were employed to examine the association between blood Cd levels and mortality rate. Stratified analyses were performed to assess the robustness of the results. Statistical analyses were performed using R software (version 4.3.1) and the Free Statistics software version 1.8. Statistical significance was set at *p* < 0.05.

## 3 Results

### 3.1 Participant characteristics

A total of 13,450 NAFLD patients were included in our analysis, with male participants accounting for 43%. Table 1 describes the patient characteristics stratified by tertiles of blood Cd levels. Compared to the T1 group, participants in the T3 group (higher blood Cd levels) were more frequently male, non-Hispanic white, widowed/divorced/separated, with a PIR < 1, smokers with higher cotinine levels, drinkers, hypertension, higher WBC, higher energy intake, and lower FIB-4 index. Additionally, the T3 group had lower average age, education level, BMI, and moderate physical activity. As of December 31, 2019, 2,261 (16.8%), 632 (4.7%), and 516 (3.8%) participants had died from all-cause, cardiovascular, and cancer-related causes, respectively.

**Table 1:** Participants baseline characteristics by blood cadmium tertiles.

Variables	Blood cadmium				*p*
	Total (*n* = 13,450)	T1 (*n* = 4,279)	T2 (*n* = 4,614)	T3 (*n* = 4,557)	
Age, Mean ± SD, years	52.0 ± 16.5	50.1 ± 16.4	54.4 ± 16.4	51.5 ± 16.5	<0.001
Gender, *n* (%)					<0.001
Male	5,781 (43.0)	2,051 (47.9)	1,768 (38.3)	1,962 (43.1)	
Female	7,669 (57.0)	2,228 (52.1)	2,846 (61.7)	2,595 (56.9)	
Race, *n* (%)					<0.001
Mexican American	2,931 (21.8)	1,057 (24.7)	1,116 (24.2)	758 (16.6)	
Other Hispanic	1,049 (7.8)	411 (9.6)	361 (7.8)	277 (6.1)	
Non-Hispanic White	6,038 (44.9)	1,749 (40.9)	2,005 (43.5)	2,284 (50.1)	
Non-Hispanic Black	2,714 (20.2)	841 (19.7)	900 (19.5)	973 (21.4)	
Other Race	718 (5.3)	221 (5.2)	232 (5)	265 (5.8)	
Education, *n* (%)					<0.001
Less than high school	3,971 (29.5)	1,081 (25.3)	1,384 (30)	1,506 (33)	
High school	3,352 (24.9)	981 (22.9)	1,105 (23.9)	1,266 (27.8)	
More than high school	6,127 (45.6)	2,217 (51.8)	2,125 (46.1)	1,785 (39.2)	
Marital status, *n* (%)					<0.001
Married/living with partner	8,260 (61.4)	2,830 (66.1)	2,906 (63)	2,524 (55.4)	
Widowed/divorced/separated	3,281 (24.4)	843 (19.7)	1,141 (24.7)	1,297 (28.5)	
Never married	1,772 (13.2)	573 (13.4)	511 (11.1)	688 (15.1)	
Unknown	137 (1.0)	33 (0.8)	56 (1.2)	48 (1.1)	
Poverty income ratio, *n* (%)					<0.001
<1	2,468 (18.3)	668 (15.6)	726 (15.7)	1,074 (23.6)	
1–3	5,396 (40.1)	1,645 (38.4)	1,837 (39.8)	1,914 (42)	
≥3	4,488 (33.4)	1,638 (38.3)	1,633 (35.4)	1,217 (26.7)	
Unknown	1,098 (8.2)	328 (7.7)	418 (9.1)	352 (7.7)	
Body mass index, Mean ± SD, Kg/m^2^	32.9 ± 5.9	33.2 ± 6.4	33.0 ± 5.8	32.6 ± 5.6	<0.001
Diabetes, *n* (%)					0.186
Yes	3,255 (24.2)	1,052 (24.6)	1,143 (24.8)	1,060 (23.3)	
No	10,195 (75.8)	3,227 (75.4)	3,471 (75.2)	3,497 (76.7)	
Moderate physical activity, *n* (%)					<0.001
Yes	5,229 (38.9)	1,793 (41.9)	1,835 (39.8)	1,601 (35.1)	
No	8,024 (59.7)	2,452 (57.3)	2,710 (58.7)	2,862 (62.8)	
Unknown	197 (1.5)	34 (0.8)	69 (1.5)	94 (2.1)	
Smoking status, *n* (%)					<0.001
Yes	7,141 (53.1)	1,417 (33.1)	2,114 (45.8)	3,610 (79.2)	
No	6,309 (46.9)	2,862 (66.9)	2,500 (54.2)	947 (20.8)	
Cotinine, Median (IQR), ng/mL	0.1 (0,36.2)	0 (0,0.1)	0 (0,0.3)	102 (0.1,238)	<0.001
Drinking status, *n* (%)					<0.001
Yes	9,597 (71.4)	3,037 (71)	3,108 (67.4)	3,452 (75.8)	
No	3,853 (28.6)	1,242 (29)	1,506 (32.6)	1,105 (24.2)	
Hypertension, *n* (%)					<0.001
Yes	5,861 (43.6)	1,716 (40.1)	2,087 (45.2)	2,058 (45.2)	
No	7,589 (56.4)	2,563 (59.9)	2,527 (54.8)	2,499 (54.8)	
WBC, 1,000 cells/uL	7.5 ± 2.2	7.3 ± 1.9	7.3 ± 2.2	8.0 ± 2.3	<0.001
Energy intake, Mean ± SD, kcal	2046.2 ± 958.2	2105.5 ± 946.0	1982.3 ± 910.2	2055.1 ± 1011.9	<0.001
FIB-4, *n* (%)					<0.001
≤2.67	5,424 (40.3)	1,894 (44.3)	1,617 (35)	1,913 (42)	
>2.67	8,026 (59.7)	2,385 (55.7)	2,997 (65)	2,644 (58)	
All-cause mortality, *n* (%)					<0.001
No	11,189 (83.2)	3,873 (90.5)	3,777 (81.9)	3,539 (77.7)	
Yes	2,261 (16.8)	406 (9.5)	837 (18.1)	1,018 (22.3)	
Cardiovascular mortality, *n* (%)					<0.001
No	12,818 (95.3)	4,160 (97.2)	4,375 (94.8)	4,283 (94)	
Yes	632 (4.7)	119 (2.8)	239 (5.2)	274 (6)	
Cancer mortality, *n* (%)					<0.001
No	12,934 (96.2)	4,189 (97.9)	4,437 (96.2)	4,308 (94.5)	
Yes	516 (3.8)	90 (2.1)	177 (3.8)	249 (5.5)	

T1: <0.33 μg/L, T2: 0.33–0.55 μg/L, T3: ≥0.55 μg/L.

### 3.2 Associations between blood Cd and all-cause mortality

During a mean follow-up time of 137.3 months, 2,261 patients died from all causes. Patients were categorized into tertiles based on blood Cd levels: T1 (<0.33 μg/L), T2 (0.33–0.55 μg/L), and T3 (≥0.55 μg/L), with 406, 837, and 1,018 deaths from all causes in each group, respectively. After adjusting for various covariates, elevated blood Cd levels were strongly associated with an elevated all-cause mortality risk (Table 2).

**Table 2:** The association between blood cadmium (μg/L) and mortality in patients with NAFLD.

Blood cadmium	Crude Model [HR(95%CI)]	Model 1 [HR(95%CI)]	Model 2 [HR(95%CI)]	Model 3 [HR(95%CI)]
All-cause mortality				
Continuous^#^	2.3 (1.98,2.66)	3.77 (3.17,4.47)	3.46 (2.91,4.11)	2.91 (2.39,3.53)
Tertiles				
T1	Reference	Reference	Reference	Reference
T2	1.61 (1.43,1.81)	1.21 (1.08,1.37)	1.22 (1.09~1.38)	1.2 (1.06,1.35)
T3	2.11 (1.88,2.37)	1.82 (1.62,2.05)	1.77 (1.57~1.99)	1.59 (1.41,1.8)
*P* for trend	<0.001	<0.001	<0.001	<0.001
Cardiovascular mortality				
Continuous^#^	1.98 (1.49,2.63)	3.21 (2.3,4.49)	2.99 (2.13,4.18)	2.6 (1.8,3.76)
Tertiles				
T1	Reference	Reference	Reference	Reference
T2	1.57 (1.26,1.95)	1.15 (0.92,1.43)	1.17 (0.94~1.47)	1.18 (0.94,1.48)
T3	1.94 (1.56,2.4)	1.64 (1.32,2.03)	1.6 (1.29~2)	1.52 (1.21,1.91)
*P* for trend	<0.001	<0.001	<0.001	<0.001
Cancer mortality				
Continuous^#^	3.05 (2.27,4.1)	5.15 (3.67,7.22)	4.96 (3.52,6.99)	3.79 (2.54,5.65)
Tertiles				
T1	Reference	Reference	Reference	Reference
T2	1.55 (1.2,2)	1.22 (0.95,1.58)	1.23 (0.95,1.59)	1.16 (0.9,1.5)
T3	2.35 (1.85,2.99)	2.11 (1.66,2.7)	2.08 (1.63,2.65)	1.71 (1.32,2.21)
*P* for trend	<0.001	<0.001	<0.001	<0.001

Crude Model: unadjusted. Model 1: adjusted for age, gender, race, and education. Model 2: adjustments for model 1 plus marital status, poverty income ratio, body mass index, and moderate physical activity. Model 3: adjustments for model 2 plus Smoking status, Cotinine, Drinking status, Hypertension, Diabetes, WBC, Energy, and FIB-4. ^#^Blood cadmium concentrations underwent logarithmic transformation. T1: <0.33 μg/L, T2: 0.33–0.55 μg/L, T3: ≥0.55 μg/L.

**Figure 2:**
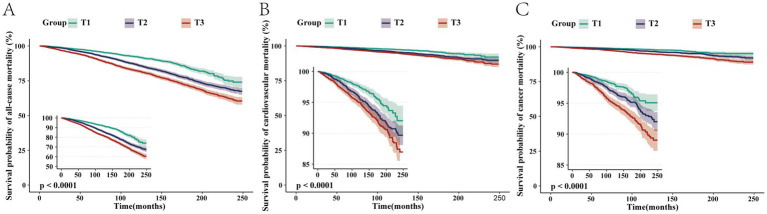
Kaplan–Meier survival curves depicting the association between blood cadmium concentrations in the T1, T2, and T3 groups with all-cause **(A)**, cardiovascular **(B)**, and cancer **(C)** mortalities in patients with NAFLD.

**Figure 3:**
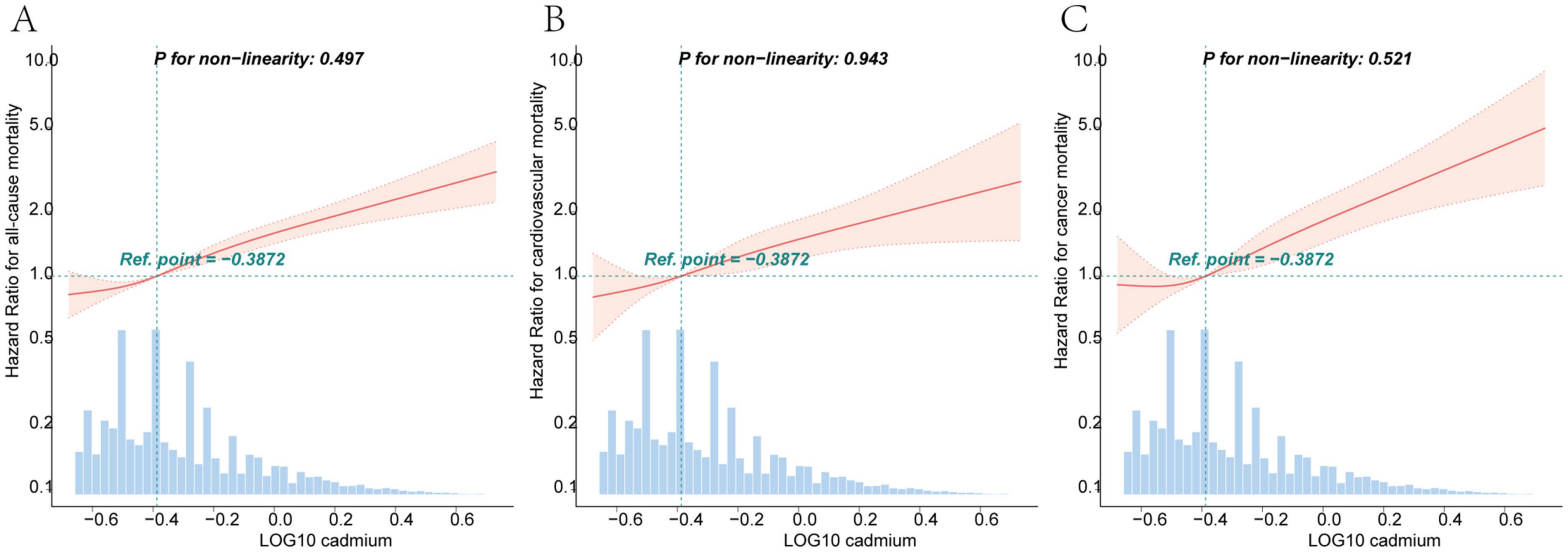
Dose–response curves of the relationship between log-transformed blood cadmium concentrations and the HRs for all-cause **(A)**, cardiovascular **(B)**, and cancer **(C)** mortalities. All models were adjusted by age, gender, race, education, marital status, poverty income ratio, body mass index, moderate physical activity, smoking status, cotinine, drinking status, hypertension, diabetes, WBC, energy intake and FIB-4.

After adjusting for all covariates, for each unit increase in log-transformed blood cadmium concentration, all-cause mortality risk in patients increased by 191% (HR = 2.91, 95% CI: 2.39–3.53). Compared to the T1 group, the T3 group exhibited a 59% increase in mortality risk (HR = 1.59, 95% CI: 1.41–1.8). Kaplan–Meier curves demonstrated that with longer follow-up, the cumulative survival rate of patients in the T3 group was significantly lower than that in the T1 group (Figure 2A). The RCS plot indicated a clear linear dose–response relationship between blood Cd concentration and all-cause mortality (nonlinear *p* = 0.497). When the HR is 1, the log-transformed blood cadmium concentration is −0.3872, which corresponds to a blood cadmium concentration of 0.415 μg/L at this point (Figure 3A).

### 3.3 Associations between blood Cd and cardiovascular mortality

In the T1, T2, and T3 groups, there were 119, 239, and 274 cardiovascular deaths, respectively. After adjusting for various covariates, elevated blood Cd levels were significantly linked to an increased risk of cardiovascular mortality (Table 2).

After adjusting for all covariates, for each unit increase in log-transformed blood Cd concentration, cardiovascular mortality risk increased by 160% (HR = 2.6, 95% CI: 1.80–3.76). Compared to the T1 group, the T3 group had a 59% higher risk of mortality (HR = 1.59, 95% CI: 1.41–1.8). Kaplan–Meier curves demonstrated that with longer follow-up, the cumulative survival rate of patients in the T3 group was significantly lower than that in the T1 group (Figure 2B). The RCS plot indicated a clear linear dose–response relationship between blood Cd concentration and cardiovascular mortality (nonlinear *p* = 0.943). When the HR is 1, the log-transformed blood cadmium concentration is −0.3872, which corresponds to a blood cadmium concentration of 0.415 μg/L at this point (Figure 3B).

### 3.4 Associations between blood Cd and cancer mortality

In the T1, T2, and T3 groups, there were 90, 177, and 249 cancer-related deaths, respectively. After adjusting for various covariates, elevated blood Cd levels were closely associated with an increased risk of cancer mortality (Table 2).

After adjusting for all covariates, for each unit increase in log-transformed blood Cd concentration, the risk of cancer mortality in patients increased by 279% (HR = 3.79, 95% CI: 2.54–5.65). Compared to the T1 group, the T3 group had a 75% higher risk of mortality (HR = 1.71, 95% CI: 1.32–2.21). Kaplan–Meier curves demonstrated that with longer follow-up, the cumulative survival rate of patients in the T3 group was significantly lower than that in the T1 group (Figure 2C). The RCS plot indicated a clear linear dose–response relationship between blood Cd concentration and cancer mortality (nonlinear *p* = 0.521). When the HR is 1, the log-transformed blood cadmium concentration is −0.3872, which corresponds to a blood cadmium concentration of 0.415 μg/L at this point (Figure 3C).

### 3.5 Subgroup analysis

**Figure 4:**
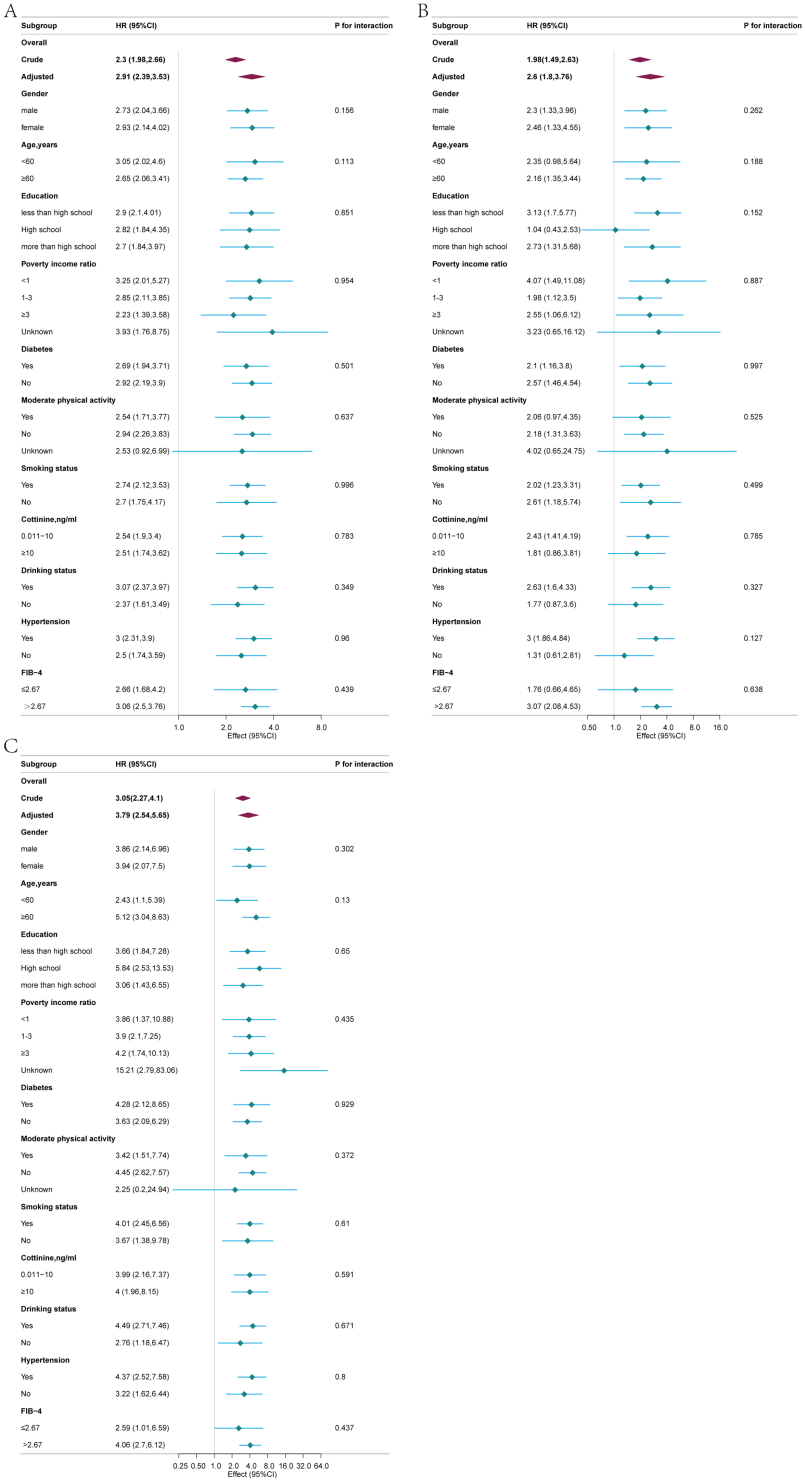
Associations between blood cadmium concentration and all-cause **(A)**, cardiovascular **(B)**, and cancer **(C)** mortalities in different subgroups, adjusted for age, gender, race, education, marital status, poverty income ratio, body mass index, moderate physical activity, smoking status, cotinine, drinking status, hypertension, diabetes, WBC, energy intake and FIB-4.

In the subgroup analyses, a robust positive association was observed between blood Cd concentration and all-cause, cardiovascular, and cancer mortality rates (Figure 4). Stratified analysis did not reveal significant interactions (*p* > 0.05). For overall mortality, there was a trend indicating a greater adverse impact of blood cadmium on prognosis in patients with advanced fibrosis (FIB-4 index ≤ 2.67 vs. FIB-4 index > 2.67: 2.66 [1.68, 4.20] vs. 3.06 [2.50, 3.76]), although the difference was not statistically significant (*p* = 0.439). Regarding cardiovascular mortality, a similar trend was observed in the advanced fibrosis subgroup, with blood cadmium showing a greater adverse effect on prognosis (FIB-4 index ≤ 2.67 vs. FIB-4 index >2.67: 1.76 [0.66, 4.65] vs. 3.07 [2.08, 4.53]), but again, the difference was not statistically significant (*p* = 0.638). For cancer mortality, there was also a trend suggesting a more pronounced adverse effect of blood cadmium on prognosis in the advanced fibrosis subgroup (FIB-4 index ≤ 2.67 vs. FIB-4 index > 2.67: 2.59 [1.01, 6.59] vs. 4.06 [2.70, 6.12]), although the difference did not reach statistical significance (*p* = 0.437).

## 4 Discussion

Our large prospective cohort study reveals a significant positive correlation between blood Cd levels and all-cause, cardiovascular, as well as cancer-related mortality in patients with NAFLD, even after adjusting for multiple covariates. Subgroup analyses corroborate the robustness of these findings. Furthermore, a significant linear dose–response relationship was observed between blood cadmium levels and mortality, with a threshold of 0.415 μg/L associated with a markedly increased risk of all-cause, cardiovascular, and cancer mortality. This indicates that NAFLD patients should aim to maintain blood cadmium levels below 0.415 μg/L. Notably, the impact of blood Cd concentration on all-cause, cardiovascular, as well as cancer-related mortality tends to be amplified in populations with advanced liver fibrosis, although the differences were not statistically significant.

Cd, a toxic heavy metal widely present in the environment, is classified as a Group 1 carcinogen. Human exposure to cadmium mainly occurs through food, tobacco smoke, and occupational exposure (28). Previous studies have linked high blood cadmium levels to increased mortality in the general U.S. population, older adults, postmenopausal women, patients with type 2 diabetes, individuals with hypertension, rheumatoid arthritis, chronic obstructive pulmonary disease, and patients with chronic kidney disease (15–17, 29–33). Our study provides reliable evidence for the association between blood Cd levels and increased all-cause, cardiovascular, and cancer mortality in patients with NAFLD.

Animal experiments have demonstrated that Cd exposure exacerbates hepatic steatosis induced by a high-fat diet, primarily through the induction of oxidative stress, inflammatory responses, cell signaling, and lipid metabolism (12, 34–38). First, cadmium promotes the production of reactive oxygen species (ROS), which enhances oxidative stress. This, in turn, leads to lipid peroxidation and hepatic steatosis. ROS can also damage DNA and proteins, resulting in hepatocyte apoptosis (14, 20, 39). Second, cadmium induces the production of inflammatory factors, such as tumor necrosis factor-alpha (TNF-*α*) and interleukin-6 (IL-6), in hepatocytes, leading to an inflammatory response in the liver. Chronic inflammation subsequently results in liver fibrosis (35). Additionally, cadmium may activate the NF-κB and MAPK signaling pathways, promoting fibroblast proliferation and collagen deposition, thereby facilitating the progression of hepatic fibrosis (40). Finally, cadmium-induced interference with lipid metabolism leads to increased fatty acid synthesis and decreased oxidation, resulting in the accumulation of excess fatty acids in hepatocytes, which contributes to steatosis (41).

Cd may contribute to atherosclerosis through oxidative stress, inflammation, and endothelial cell damage (42). It may also elevate blood pressure through vascular effects, inflammation, and blockade of calcium signaling pathways, thereby increasing the cardiovascular mortality rate in NAFLD patients (43).

Previous studies have shown controversial results regarding the association between Cd exposure and cancer risk (44–49). A recent review summarizing epidemiological and laboratory findings showed that Cd is a multi-organ carcinogen, with its exposure linked to tumors in the lungs, kidneys, pancreas, and breasts, as well as the progression of hepatocellular carcinoma (50). Our study on the NAFLD population confirmed a positive correlation between blood Cd levels and cancer mortality.

These findings have vital clinical and medical implications. Elevated blood Cd levels can serve as predictive biomarkers of mortality in patients with NAFLD. Furthermore, a interventional study conducted in cadmium-contaminated areas demonstrated that participants consuming low-cadmium rice exhibited better blood pressure and renal function compared to those consuming rice with high levels of cadmium contamination (51). Our findings may enhance awareness of Cd exposure in patients with NAFLD and help healthcare professionals implement timely interventions to prevent disease progression. Finally, given the toxic nature of cadmium and its role in exacerbating liver damage, public health measures such as stricter regulation of industrial emissions, improved food safety standards, and targeted interventions in occupational settings could significantly reduce cadmium exposure.

Our study has several strengths. First, this study has the largest sample size to date examining the association between blood Cd levels and mortality in patients with NAFLD. Furthermore, this was a prospective cohort study that considered various potential confounding factors, including smoking status and blood cotinine levels, and conducted a stratified analysis, enhancing the credibility of our results. Additionally, we considered cause-specific mortality outcomes, making our study results more precise and practically valuable.

However, this study has some limitations. Occupational Cd exposure and dietary habits, which are known potential confounders, were not fully accounted for in this analysis due to data limitations. Future studies should aim to include these factors to reduce potential bias. Additionally, the predictive performance of individual factors on prognosis is limited. Future research could combine blood cadmium concentration with other environmental and dietary factors using machine learning algorithms to establish predictive models, which represents one of our future research directions. Finally, due to the limitations of the NHANES database, repeated measurements and long-term tracking of blood cadmium concentrations were not conducted; addressing this issue could potentially provide more accurate prognostic estimates.

## 5 Conclusion

In conclusion, our study demonstrated that blood Cd levels are independent risk factors for all-cause, cardiovascular, and cancer-related mortality in individuals with NAFLD. However, the molecular mechanisms by which blood cadmium influences the prognosis of patients with NAFLD require further investigation.

## Data Availability

The datasets presented in this study can be found in online repositories. The names of the repository/repositories and accession number(s) can be found at: https://www.cdc.gov/nchs/nhanes/.
